# The prophylactic effects of a traditional Japanese medicine, goshajinkigan, on paclitaxel-induced peripheral neuropathy and its mechanism of action

**DOI:** 10.1186/1744-8069-10-61

**Published:** 2014-09-21

**Authors:** Yukiko Matsumura, Yoshihito Yokoyama, Hachidai Hirakawa, Tatsuhiko Shigeto, Masayuki Futagami, Hideki Mizunuma

**Affiliations:** Department of Obstetrics and Gynecology, Hirosaki Graduate School of Medicine, 5-Zaifu-cho, Hirosaki, Aomori, 036-8562 Japan

**Keywords:** Paclitaxel, Goshajinkigan, Peripheral neuropathy, Degeneration of the ganglion cells, TRPV4

## Abstract

**Background:**

This study aimed to evaluate the prophylactic effect of goshajinkigan (GJG) on paclitaxel (PTX)-induced neuropathy and to elucidate the mechanism of action.

**Results:**

There was a time-dependent irreversible decrease in pain threshold in PTX group. In PTX/GJG group, pain threshold showed changes in the same level as control. Electron microscope showed that although the ganglion cells of control and PTX/GJG groups were normal, degeneration of the nucleus and swelling of the mitochondria were observed in PTX group. Expression of *transient receptor potential vanilloid 4 (TRPV4)* gene in PTX group significantly increased compared with that in control and PTX/GJG groups. In *TRPV4* knock-out mice, no PTX-induced hyperalgesia was observed, and there was no significant difference in pain threshold between the 3 groups.

**Conclusions:**

These results showed that PTX induced hyperalgesia by enhancing TRPV4 expression, and suggested that GJG might alleviate hyperalgesia by preventing degeneration of the ganglion cells and suppressing TRPV4 expression.

## Background

The combination of paclitaxel and carboplatin (TC therapy) is the recommended standard chemotherapy following a surgery for epithelial ovarian cancer [1]. In order to reduce adverse effects during and after TC therapy, supportive therapies for the digestive symptoms, myelosuppression, and hypersensitivity caused by anti-cancer drugs have been invented and established [2–4]. However, measures against peripheral neuropathy (numbness) caused by paclitaxel (PTX) remain unknown. The incidence of sensory peripheral neuropathy in TC therapy was 78% [5], and worsens the rate of treatment completion and reduces quality of life (QOL) of patients. Many attempts using NSAIDs, steroids, antihistamines, tryptophan-N-formylated gramicidin (NFG), glial cell-line derived neurotrophic factor (GDNF), amifostine, gabapentin, and carbamazepine [6–8] have been conducted aiming to attenuate the drug-induced peripheral neuropathy, however, no methods have been established thus far.

Recently, the temperature-sensitive transient receptor potential (TRP) channels are shown to be involved in chemotherapy-induced neuropathic pain [9, 10]. TRP channels are group of ion channel receptors located in the cell membrane and form a large channel group consisting of 6 subfamilies and 28 channels in humans [11]. Since the discovery of a temperature-sensitive TRP channel in 1997, 9 TRP channels were observed to be temperature-sensitive [12]. Many TRP channels are present in various parts of the body and play an important role as biological sensors for receiving a number of chemical and physical stimulations [13]. For example, TRPV1 is activated by stimulations of heat, acid, capsaicin, and pain.

Goshajinkigan (GJG) is a traditional Japanese medicine, comprising 10 types of crude drugs, rehmannia root, discorea rhizome, cornus fruit, hoelen, alisma rhizome, moutan bark, cinnamon bark, aconite root, achyranthes root, and plantago seed, and has been used for pain control in the lower extremities, numbness, blurred vision, difficulty in urination, and edema [14]. GJG has been shown to increase peripheral blood flow and exhibit analgesic effects by increasing NO production [15], and is shown to be effective for a treatment of numbness caused by diabetes mellitus and cancer chemotherapy [16], although the mechanism of action of GJG is unknown. However, it has recently been suggested that TRP channels are involved in the effect of GJG [17].

The present study aimed to clarify whether GJG is effective to reduce anti-cancer drugs induced neuropathy and to elucidate its mechanisms in conjunction with TRP.

## Results

### Effect of GJG on mechanical allodynia in PTX-treated rats

**Figure 1:**
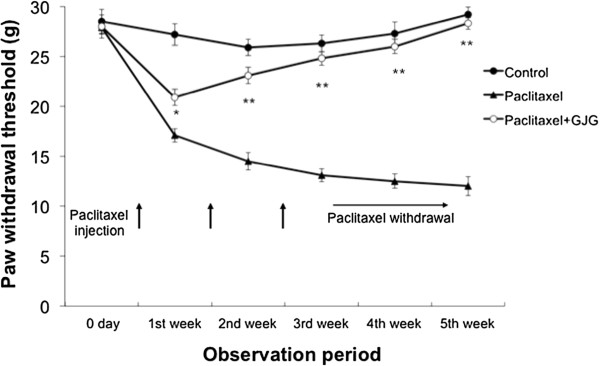
**Comparison of the pain threshold.** The pain threshold was irreversibly decreased in PTX group. The pain threshold of the PTX + GJG group was comparable with that of control group. *P < 0.005, **P < 0.0001; PTX vs. PTX + GJG.

The pain threshold in the PTX group showed a significant decrease at the 1^st^ week of PTX administration and stayed at decreased levels even after PTX withdrawal, suggesting that PTX increased the sensitivity to pain and that the effect of PTX last longer (Figure 1). The pain threshold of PTX + GJG group, on the other hand, showed a temporally decrease at 1^st^ week of experiment but thereafter increased and returned to similar levels of control groups, suggesting that GJG prevented the effect of PTX on pain threshold (Figure 1). From day 2 of administration of PTX, a significant difference in the pain threshold between the PTX and PTX + GJG groups was observed (Figure 1). The administration of PTX and GJG did not result in adverse reactions such as weight loss and self-harming.

### Electron microscope findings in dorsal root ganglion cells

**Figure 2:**
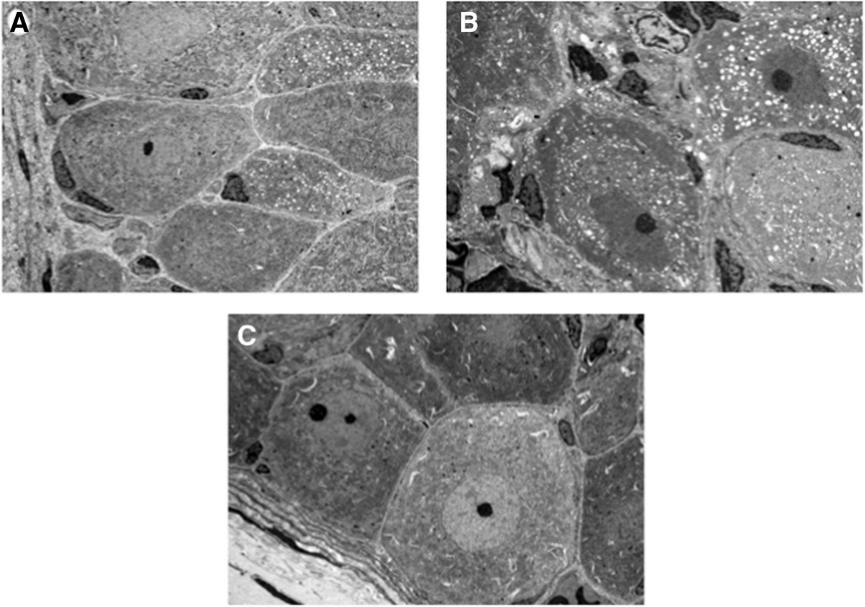
**Electron microscope findings of the dorsal root ganglion cell. A**. Control, magnification of 1000×. **B**. PTX, magnification of 1500×. **C**. PTX + GJG, magnification of 1000×.

In comparison with the control group, there was clear degeneration of the nucleus and swelling of the mitochondria in the PTX group, suggesting that PTX caused neurodegenerative (Figure 2). In the PTX + GJG group, however, there was no apparent degeneration of the nucleus and swelling of the mitochondria and generally showed cellular findings comparable with the control group (Figure 2).

### Screening of expression of TRP gene by DNA microarray

**Figure 3:**
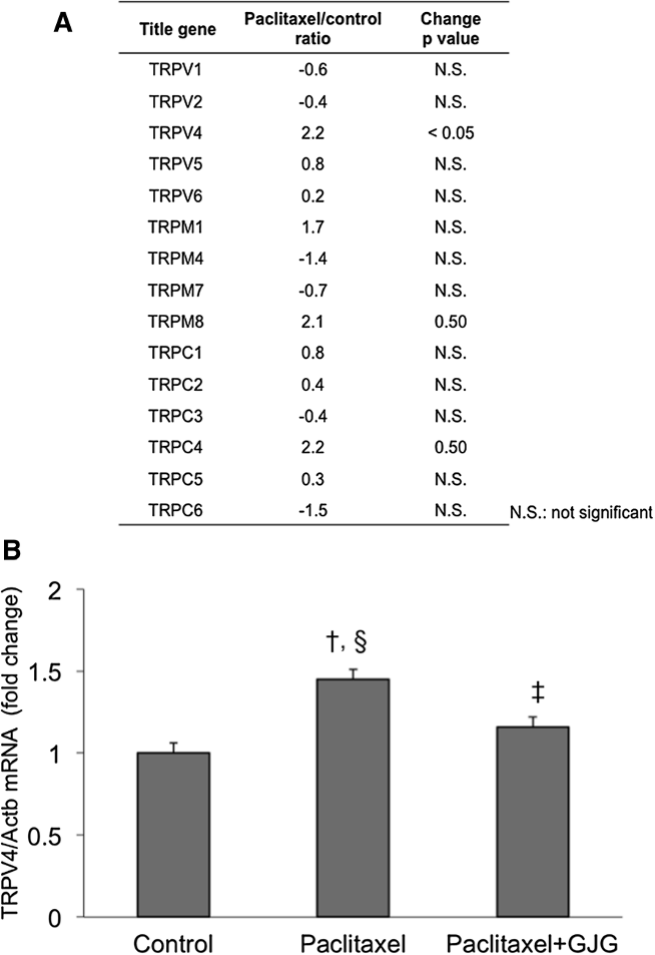
**Altered expression of TRP genes by paclitaxel. A**. Screening of expression of TRP genes by DNA microarray. **B**. TRPV4 validaton by RT-PCR. TRPV4 gene, whose change in expression was significant in DNA microarray, was determined by TaqMan Gene Expression Assay. ^†^P < 0.001, vs. control. ^§^P < 0.005, vs. PTX + GJG. ^‡^P = 0.05, vs. control.

Gene expression of TRP subtypes in the DRG cells of the control and PTX groups was compared using DNA microarray. A significant change was defined as the ratio of change in expression ≥2 and the change value ≤0.05. However, out of the 15 types of TRP subtypes that could be analyzed, only TRPV4 showed a significant change (Figure 3A).

### Validation of TRPV4 gene by RT-PCR

Gene expression of TRPV4 in the DRG, which showed a significant change, was determined by TaqMan Gene Expression Assay. PTX group showed significant increase in TRPV4 compared with the control and PTX + GJG groups (Figure 3B). In contrast, the results showed that co-administration of GJG significantly decreased gene expression of TRPV4 (Figure 3B).

### Comparison of protein expression of TRPV4 in the DRG

**Figure 4:**
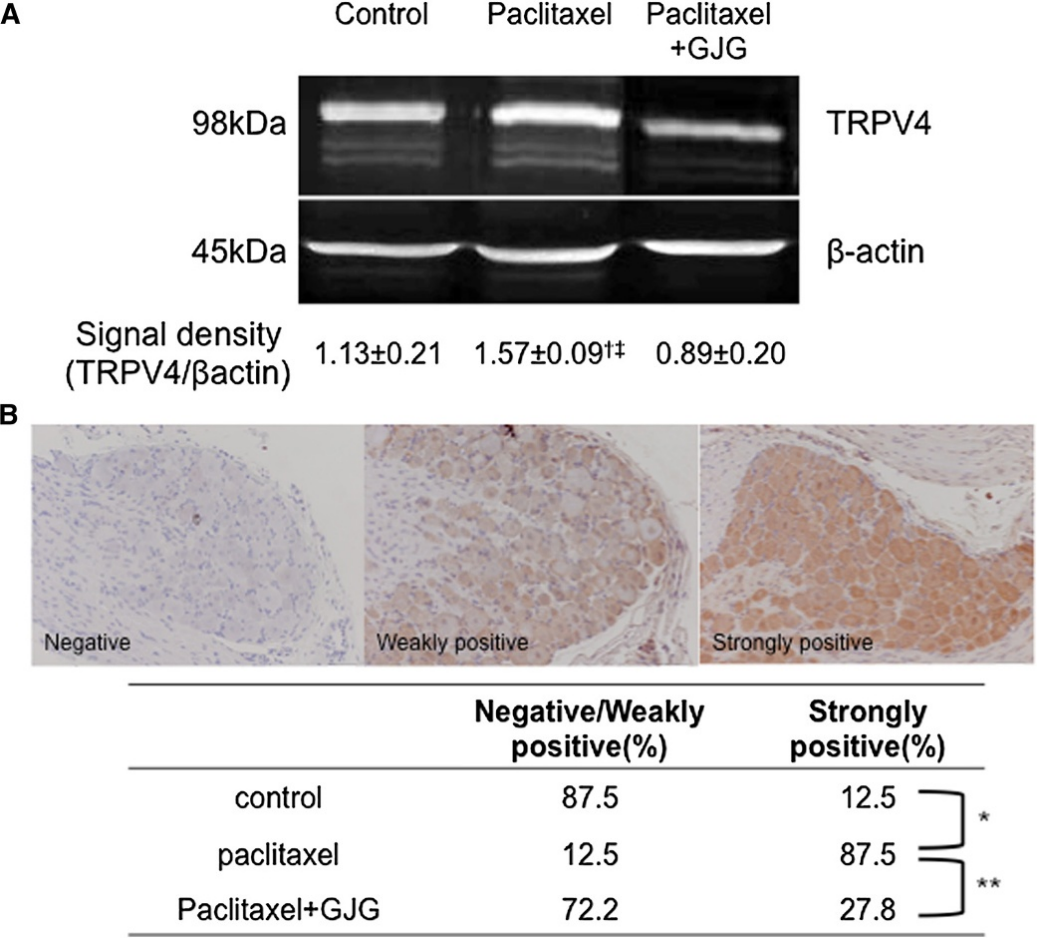
**Comparison of protein expression of TRPV4 in the dorsal root ganglions. A**. Comparison of the expression of TRPV4 by Western blot. Expression of TRPV4 was significantly increased in the PTX group and significantly suppressed by the co-administration of GJG. The experiment was repeated 3 times, and the means were expressed numerically. The figures shown are representative photographs. ^†^P < 0.05, vs. control. ^‡^P < 0.01, vs. PTX + GJG. **B**. Comparison of the expression of TRPV4 by immunohistochemistry. The expression of TRPV4 in ganglion cell was classified into negative, weakly positive, and strongly positive, as shown in the figure and the level of TRPV4 expression between the 3 groups was compared. *P < 0.005, **P < 0.01.

When the protein expression of TRPV4 in the DRG was analyzed using Western blot through signal density, the PTX group showed a significant increase compared with the control group (Figure 4A, P < 0.05), and the expression of TRPV4 was significantly suppressed in the PTX + GJG group compared with the PTX group (Figure 4A, P < 0.01). Moreover, when the expression of TRPV4 in DRG cells was classified into negative, weakly positive, and strongly positive, it was confirmed that the expression of TRPV4 in the DRG cells was significantly increased in the PTX group compared with that in the control group (Figure 4B, P < 0.005) and significantly suppressed in the PTX + GJG group (Figure 4B, P < 0.01).

### Comparison of the expression of TRPV4, integrin, and Src tyrosine kinase using tissue microarray

**Figure 5:**
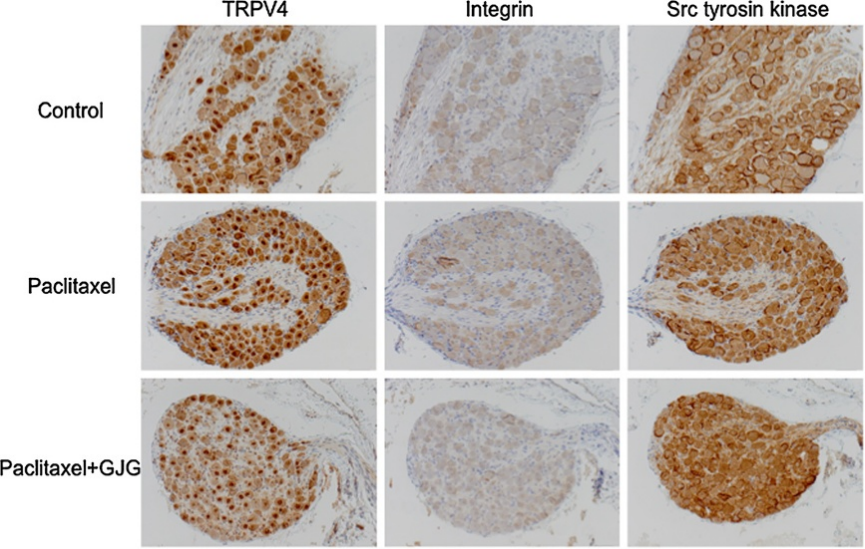
**Comparison of the expressions of TRPV4, integrin, and Src tyrosine kinase by tissue microarray.** In PTX group, TRPV4, integrin, and Src tyrosine kinase were more strongly expressed in the nucleus, cytoplasm, and cell membrane in ganglion cell compared with the control group. In PTX + GJG group, no clear change in the expression of Src tyrosine kinase and integrin was noted, but the expression of TRPV4 in the nucleus and cytoplasm was clearly decreased compared with PTX group.

For TRPV4 to intracellularly communicate sensory stimulations, it is essential for integrin and Src tyrosine kinase to bind to the stimulation receptor TRPV4. Thus, the expressions of TRPV4, integrin, and Src tyrosine kinase in the DRG were simultaneously compared using tissue microarray. In the PTX group, TRPV4, integrin, and Src tyrosine kinase were more strongly expressed in the nucleus, cytoplasm, and cell membrane of the ganglion cells compared with the control group (Figure 5). A characteristic finding in the PTX + GJG group was that the expression of TRPV4 in the nucleus and cytoplasm was clearly decreased compared with that in the PTX group (Figure 5). Meanwhile, between the PTX group and PTX + GJG groups, no clear change in expression was found in Src tyrosine kinase and integrin (Figure 5).

### The shift in pain threshold induced by PTX in TRPV4 knock-out mice

**Figure 6:**
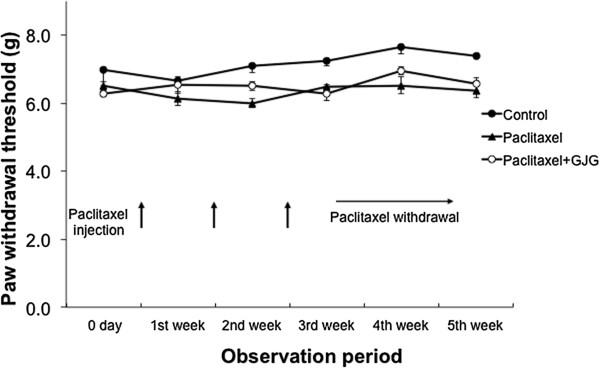
**PTX-induced shift in the pain threshold in TRPV4 knock-out mice.** PTX did not induce hyperalgesia, and there was no significant difference in the pain threshold between the 3 groups.

There was no decrease in pain threshold following the administration of PTX. There was no significant difference in the pain threshold between the 3 groups (Figure 6).

## Discussion

This study showed that the administration of PTX caused a significant and irreversible decrease in pain threshold (increase in hyperalgesia), and that administration of GJG blocked the effect of PTX. PTX is a mitotic inhibitor used for cancer chemotherapy as it acts by targeting the microtubules necessary for the transport of chromosomes during cell division [18]. It has been reported in the PTX-induced pain model that PTX treatment causes changes in nerve axon diameter, disorientation of microtubules, and mitochondrial swelling [19]. The result of the present study has confirmed electron-microscopically that PTX administration induced neural degeneration such as the nucleus and mitochondrial swelling in the DRG cells. In addition, present study showed for the first time that GJG administration decreases pain threshold by protecting the nerve cells from degeneration.

TRP channels are group of ion channel receptors located in the cell membrane and form a large channel group consisting of 6 subfamilies and 28 channels in humans [11]. Many TRP channels are present in various parts of the body and play an important role as biological sensors for receiving a number of chemical and physical stimulations [13]. Kato et al. have shown that oxaliplatin caused hypersensitivity to cold sensation and that GJG reduced then oxaliplatin-induced hypersensitivity and its effect would be related to suppression of the expression of TRPM8 and TRPA1 in the DRG of rats [17]. As shown in Figure 3A, however, the present study has shown that TRPV4 was involved in PTX-induced hyperalgesia. TRPV4 is a molecule widely involved in the development of neuropathic pain [20] and elicits its action through formation of a complex with integrin or Src tyrosine kinase [20]. In general, it is said that TRPV4 was mainly distributed in the skin but a little in the sensory nerves [21]. However, the present study clearly demonstrated that TRPV4 is present in sensory nerves and is induced in the DRG cells in response to administration of PTX. In addition, integrin and Src tyrosine kinase also expressed at high levels in response to administration of PTX, strongly suggesting that TRPV4 in DRG cells is involved in PTX-induced hypersensitivity to pain sensation. As shown in Figure 6, *TRPV4* knock-out mice did not show any decrease in pain threshold as a result of PTX administration, confirming that TRPV4 is involved in PTX-induced hyperalgesia. In addition, induction of TRPV4 and its complex with integrin and Src tyrosine kinase showed no apparent increase upon co-administration with GJG, suggesting that the action of GJG is to suppress the expression of TRPV4, integrin and Src tyrosine kinase complex.

GJG is a compound consisting from 10 components and each component has its own pharmacologic action [22–24], but it is thought that co-administration of 10 components exerts most suitable effects as a clinical regimen. Indeed, it is shown that GJG is effective against “numbness” caused by diabetic neuropathy [14]. In addition, laboratory studies have shown that GJG exerts many pharmacological effects. For example, GJG suppresses the release of pain-transmitting substance, increases NO production mediated by bradykinin B2 receptor and muscarinic acetylcholine receptor [15] and reduces reactive oxygen species production [25]. Because PTX therapy accumulates of hydrogen peroxide [26, 27] and antioxidant such as N-acetylcysteine can alter the cytotoxicity of PTX [28] and PTX-induced mechanical hyperalgesia [29], it is presumed that antioxidant action of GJG is likely be involved in protecting PTX-inducing neural damage. Materazzi et al. demonstrated that an antioxidant, glutathione, suppresses PTX-induced expression of TRPV4 in the sensory nerve [30], which strongly supports our presumption.

In thermoregulatory system, one of the ion channel receptors called TRPM8 plays an important role [31]. Peripheral hypersensitivity to cold sensations is commonly associated with oxaliplatin-induced peripheral neurotoxicity, and possible involvement of TRPM8 is demonstrated [32]. Of interest, Ushio et al. showed in oxaliplatin-treated animals that GJG reduced hypersensitivity to cold stimulation but not hypersensitivity to mechanical stimulation [33]. As shown in Figure 3A, we could not find any significant change in TRPM8 gene expression in PTX administration, suggesting that oxaliplatin and PTX elicit neurotoxicity in a different manner. Nevertheless, the fact that GJG could reduce the neurotoxicity caused by oxaliplatin and PTX may suggest that GJG is useful for anti-cancer-drug induced neuropathy in clinical practice. Indeed, the efficacy of GJG on peripheral neuropathy has been accumulated in clinical studies on patients receiving chemotherapies [16, 34, 35].

In a clinical study on PTX for ovarian and endometrial cancer in patients with peripheral neuropathy, GJG was observed to stop the progression of peripheral neuropathy to a certain degree, but there was no clear improvement in symptoms compared with that in the control group [35]. As it is apparent from this study, hyperalgesia occurred in the early stages of administration of PTX and caused irreversible changes in rats. In this study, PTX-induced peripheral neuropathy could be prevented by administering GJG prior to the administration of PTX. It is possible that irreversible peripheral neuropathy also occurs in humans in early stages of administration of PTX. When verifying the prophylactic effects of GJG against PTX-induced peripheral neuropathy, it is advisable that the clinical study is designed to administer GJG prior to the administration of PTX.

## Methods

### Animals

The animal experiments were conducted in accordance with the Guidelines for Animal Experimentation, Hirosaki University and the guidelines of the International Association for the Study of Pain. Eight-week-old female Fisher344 rats weighting 180–200 g and *Trpv4−/−* mice [36, 37] weighting 20–25 g were used in this study. They were group-housed in plastic cages with stainless-steel grid tops and kept in an air-conditioned room maintained at 22 ± 2°C in the Institute for Animal Experiments of Hirosaki University. Artificial light was provided on 12-hour cycle. They had free access to food and water.

### Paclitaxel-induced neuropathy model and drugs administration

PTX was obtained from Nippon Kayaku (Tokyo, Japan). PTX was diluted with sterile saline and injected intraperitoneally (i.p.) at a dose of 10 mg/kg per a week for three weeks to establish a rodent model presenting PTX-induced neuropathy [38]. GJG was obtained from TSUMURA & Co. (Tokyo, Japan). GJG was diluted with distilled water.

The experimental rodents were divided into three groups containing 5 rodents each. Control group received basal diet alone. PTX group was administered PTX at 10 mg/kg i.p. by the method mentioned above. The combination of GJG and PTX treatment group (PTX + GJG group) was given GJG into the esophagus through a per os gastric tube at a dose of 150 mg/kg once a day for 5 weeks from a week before PTX administration and was given paclitaxel in the same way as administered for PTX group. The single dose of GJG administered to the rodents conforms to that at which a patient weighing 50 kg is given per a day.

### Behavioral analysis

Behavioral tests were done in a quiet, temperature-controlled room between 1 p.m. and 4 p.m. and were carried out by an inspector who was not informed of treatment group. PTX-induced mechanical allodynia and hyperalgesia were measured with an automated von Frey testing device, a dynamic plantar esthesiometer (Ugo Basile, Italy). Rodents were placed in clear plastic box (110 × 180 × 140 mm) on wire mesh floor and allowed to acclimatize for 1 hour. The touch-stimulator was moved below the rodent right hind paw. The actuator filament (0.5 mm diameter) moved upward increasing force (force increasing rate: 5 g/s, over a 20 s period) until the rodents removed their paw. When the paw was withdrawn, the actuator moving was stopped and the device was recorded the force at which the rodent withdrew its paw. The examination was performed once before PTX administration (baseline), the second day after PTX administration and every week after PTX withdrawal. Stimulation to a rodent was applied three times at 1-2-min interval, and the average was calculated.

### Isolation of primary sensory neurons

The rats of each group were sacrificed with a high dose of i.p. sodium pentobarbital (200 mg/kg) on Day 35 after PTX administration, and dorsal root ganglia (DRG) was removed for pathological, molecular and biochemical studies. The lumbar DRG (L4 and 5) were bilaterally excised under a dissection microscope.

### Electron microscopy

The excised DRG were fixed in 2.5% glutaraldehyde solution, washed with 7.5% sucrose for 10 min, post-fixed for 2 hours in 1% osmium tetroxide, and after washed with 50% ethanol, dehydration was done continuously through a graded ethanol series. After substitution from ethanol to QY-1 (Okenshoji Co., Ltd, Tokyo, Japan), these specimens were embedded in epoxy resin (TAAB Laboratories Equipment Ltd, Berkshire, UK) and ultra-thin sections (80–100 nm) were collected on copper grids by using Ultratome® NOVA (LKB, Bromma, Sweden). Double-staining was done with uranyl acetate (for 20 min) followed by lead citrate (for 10 min). Finally, these specimens were examined and photographed with JEM 1010 (JEOL, Tokyo, Japan) equipped with Orius® SC200 CCD camera (Gatan, CA) for transmission electron microscopy at 80 kV of acceleration voltage.

### Total RNA isolation

Tissues were first homogenized by homogenizer and then by the TRIzol reagent (Invitrogen Life Technologies, Carlsbad, CA) for the total RNA extraction following the manufacturer’s instructions. The extracted total RNA was then purified using the RNeasy MiniElute Cleanup Kit column (Qiagen, Tokyo, Japan) with the DNase incubation (Qiagen). Total RNA extracted was quantified using an Eppendorf UV spectrophotometer, and the integrity of the RNA samples was controlled using Agilent 2100 Bioanalyzer (Agilent Technologies Inc, Palo Alto, CA) with the NanoDrop ND-1000 Kit (Thermo Fisher Scientific Inc., Waltham, MA). Only RNAs with OD260/OD280 > 1.8 and an RNA integrity number (RIN) > 7 were used for microarray experiments. The remaining good-quality RNAs were kept for the subsequent RT-PCR confirmative experiments.

### Microarray analyses

Each good-quality sample was hybridized to the Affymetrix HG-U133 plus 2.0 GeneChip (Affymetrix, Santa Clara, CA). This gene chip analyzes the expression level of 38,500 well-characterized human genes. First, 10 μg of the total RNA was reverse-transcribed with the SuperScript Choice System (Invitrogen) with oligo dT primers containing a T7 RNA polymerase promoter site. Then, cDNA was in vitro transcribed and labeled with biotin using the IVT labeling kit (Affymetrix) followed by the fragmentation of the biotinylated cRNA. Next, the quality of this cRNA was assessed with the Agilent 2100 Bioanalyzer. The fragmented cRNA was hybridized overnight to Affymetrix Human Genome U133APlus 2.0 Arrays and scanned following the guidelines. The chips were washed and stained using the GeneChip Fluidics Station 400 (Affymetrix) and then scanned with the GeneChip Scanner 3000 (Affymetrix). The ratio from signal intensity values of four time points was calculated. Pathways regulated at *p* < 0.05 were considered significant.

### RT-PCR

Quantitative PCR (qPCR) was utilized to evaluate gene expression of *Trpv4*. TaqMan® Gene Expression Assays (Life Technologies, Forester City, CA) was used for assay: TaqMan® probes of Trpv4 (Rn00583117_m1) and Actb (Rn00667869_m1) were used. Primers used for PCR were as follows: TRPV4, 5′-ACCAGTACTATGGCTTCTCC-3′ and 5′-AATTCCCTACTCTACCCTGC-3′. Actb, 5′-AGTCCCTTGCCATCCTAAAAGC-3′ and 5′-GGGAGAGGACTGGGCCATT-3′. Before qPCR, reverse transcription of 500 ng of total RNA was performed using SuperscriptR VILO® cDNA synthesis kit (Life Technologies), qPCR reactions were set-up in 20 μl volumes with 50-fold cDNA dilutions, 20× TaqMan® gene expression assay mix, 2× TaqMan® Universal PCR master mix II (Life Technologies), and dH_2_O. PCRs were performed in quadruplicate following the manufacture protocols on an model ABI Prism 7000 real-time PCR system (Life Technologies) using the following protocol: an initial denaturation and polymerase activation step for 10 min at 95°C, followed by 40 cycles of denaturation at 95°C for 15 s and 60°C for 1 min. Actb was used as a reference gene to normalize between samples.

### Western blot analyses

Removed DRG tissues were homogenized in PIPA buffer in ice. The homogenate was incubated overnight at 4°C followed by centrifuging at 15000 g for 15 min at 4°C. The supernatant fluids (50 μg protein) were electrophoresed through a 10% sodium dodecyl sulfate (SDS)-polyacrylamide gel and blotted as described previously [39]. The protein concentration was determined using the Bradford method. The blots were probed with following primary antibodies; TRPV4 (Alomone Labs, Jerusalem, Israel) at 1:200 or β-actin (Sigma-Aldrich, St Louis, MO) at 1:1000. The membranes probed by TRPV4 was incubated for 2 hours with anti-rabbit immunoglobulin and transferred to the avidin-biotinylated enzyme complex (VECTA Laboratories, Burlingame, CA) for 30 min. Diaminobenzidine (DAB) (Sigma-Aldrich) was used as the substrate. The membrane probed by β-actin was incubated for 1 hour with anti-mouse immunoglobulin. Quantification of the results was performed by scanning the membrane with Photoshop software (version 5.5, Adobe Systems) followed by densitometry with the public domain software, NIH Image, version 1.62.

### Immunohistochemical analyses

Six-μm sections of formalin-fixed and paraffin-embedded tissue specimens were stained by established method as described previously [39]. Sections were routinely passed through xylen and a graded alcohol and were blocked endogenous peroxidase activity by incubating sections in 3% H_2_O_2_ solutions in methanol. Sections were incubated with blocking buffer (VECTA Laboratories) for 20 min and were reacted with Anti-TRPV4 (Alomone Labs, 1:1000) for 30 min in a humidified chamber at room temperature. Next, Sections were added a biotin-labeled secondary antibody which was biotinylated anti-rabbit IgG and were incubated with the avidin-biotinylated enzyme complex (VECTA Laboratories) for 30 min. The binding sites of peroxidase were determined using DAB as the substrate. Sections were then counterstained with hematoxylin for microscopic examinations. 2 observers (Y.Y. and H.H.) independently evaluated and interpreted the results of immnohistochemical staining. Interpretation of the immunohistochemical staining was classified into three categories based on staining intensity (negative, weekly positive, or strongly positive).

### Tissue microarray

For the tissue microarray (TMA), hematoxylin and eosin- stained sections were used to define the ganglion cells and preserved DRG from the rats used in this experiment. One ganglion representative 3 mm core was obtained from each rat and inserted in a grid pattern into a recipient paraffin block using a tissue arrayer (JF-4 Pathology Institute Corp, Toyama, Japan). Sections (2 μm) were then cut from each TMA block and stained imuunohistochemically with anti-TRRV4 (Alomone Labs, 1:1000), anti-integrin (Alomone Labs, 1:200) and ant-Src tyrosine kinase (Epitomics Inc. Burlingame, CA, 1:250).

### TRPV4 knockout mice

*Trpv4−/−* mice were generated with a C57BL/6 background as described previously [36, 37]. Adult mice from generations N8-N10 were used in this study. They were divided into three groups containing 5 mice each. PTX-induced mechanical allodynia and hyperalgesia were compared among the 3 groups of control, PTX, and PTX + GJG. PTX and GJG were given in the same way as mentioned above.

### Statistical analysis

Statistical analyses were carried out by Student’s t-test, Chi square test or Fisher’s exact probability test. A result was deemed significant at *P* < 0.05.
